# Sex-Related Differences in Short-Term Prognosis in Patients with Acute Myocardial Infarction-Related Cardiogenic Shock Receiving Impella Support in Japan: From the J-PVAD Registry

**DOI:** 10.3390/medicina59071208

**Published:** 2023-06-27

**Authors:** Makiko Nakamura, Teruhiko Imamura, Hiroshi Ueno, Koichiro Kinugawa, J-PVAD Investigators

**Affiliations:** Second Department of Internal Medicine, University of Toyama, 2630 Sugitani Toyama, Toyama 930-0194, Japan; nakamura@med.u-toyama.ac.jp (M.N.);

**Keywords:** mechanical circulatory support, gender difference, unloading, acute heart failure

## Abstract

*Background and Objectives:* Sex-specific outcome in patients with acute myocardial infarction-related cardiogenic shock (AMI-CS) receiving temporary mechanical circulatory support remains controversial. *Materials and Methods:* Patients with AMI-CS who received Impella support were prospectively enrolled in the Japanese registry for Percutaneous Ventricular Assist Device. Patients enrolled between January 2021 and December 2022 were considered to be eligible. Patients with out-of-hospital cardiac arrest and those without revascularization were excluded. The sex disparity in the 30-day survival after the initiation of Impella support was evaluated. *Results:* A total of 924 patients (median age 73 years; 21% female) were included. Female patients were older and had a smaller physiques than male patients (*p* < 0.05 for both). Female sex was significantly associated with a higher 30-day mortality after adjustment for four other potential confounders with a hazard ratio of 1.365 (95% confidence interval 1.026–1.816, *p* = 0.0324). In the female cohort, patients who received Impella prior to revascularization (N = 138) had a greater survival rate compared to those who received Impella after revascularization (68.1% versus 44.8%, *p* = 0.0015). *Conclusions:* Among the patients with AMI-CS who received Impella support and underwent revascularization, female sex was independently associated with a lower 30-day survival. For female patients, early initiation of Impella support prior to revascularization may improve their clinical outcomes.

## 1. Introduction

Cardiogenic shock (CS) is defined as a critical state of end-organ hypoperfusion due to primary pump failure, nearly 80% of which is caused by acute myocardial infarction (AMI) [1]. In patients with AMI-associated CS (AMI-CS), in addition to primary pump failure, systemic inflammation and hypoxemia are also implicated, leading to further hemodynamic deterioration and progression of end-organ failure [2]. The CULPRIT-SHOCK trial demonstrated that immediate revascularization of the target coronary artery reduced 30-day mortality from 51.6% to 43.3% [2].

Temporary mechanical circulatory support (MCS) devices, including intra-aortic balloon pump (IABP), percutaneous left ventricular assist device (Impella), and extracorporeal membrane oxygenation (ECMO), have recently been used to support hemodynamics and maintain end-organ function in carefully selected patients. However, there are conflicting data on the clinical outcomes, and the optimal device selection and timing of temporary MCS for patients with AMI-CS remains controversial [2]. In particular, sex differences in the clinical impact of temporary MCS in this cohort remain uncertain [3,4,5,6].

Impella is an emerging temporary MCS that has advantages over IABP in improving hemodynamics, unloading the left ventricle, and preserving coronary circulation. The clinical use of Impella is also increasing in Japan in various clinical scenarios, especially in AMI-CS [7]. However, there are few studies evaluating the sex disparity in this clinical scenario. Of note, Japanese female patients are older and have smaller physiques, both of which might affect the clinical impact of Impella differently from other countries, given the size mismatch between the device and cardiovascular anatomy [8,9].

The Japanese registry for Percutaneous Ventricular Assist Device (J-PVAD) is a multi-center prospective observational registry for patients who received Impella-integrated MCS in Japan [7,10]. In this study, we evaluated the sex-related differences on the short-term clinical outcomes in patients with AMI-CS receiving Impella using J-PVAD registry data.

## 2. Methods

### 2.1. Study Design and Patient Selection

The J-PVAD is a multicenter, prospective, observational registry that was initiated in October 2017. The study was registered with the University hospital Medical Information Network Clinical Trials Registry in Japan (ID: UMIN000033603). All patients with drug-refractory acute heart failure (i.e., CS) who underwent Impella placement were enrolled in the J-PVAD. The registry complied with the Declaration of Helsinki and was approved by the Central Institutional Review Board of Osaka University (Approval No. 17232). Individual patient data were collected by participating investigators and stored directly in a central electronic database.

**Figure 1:**
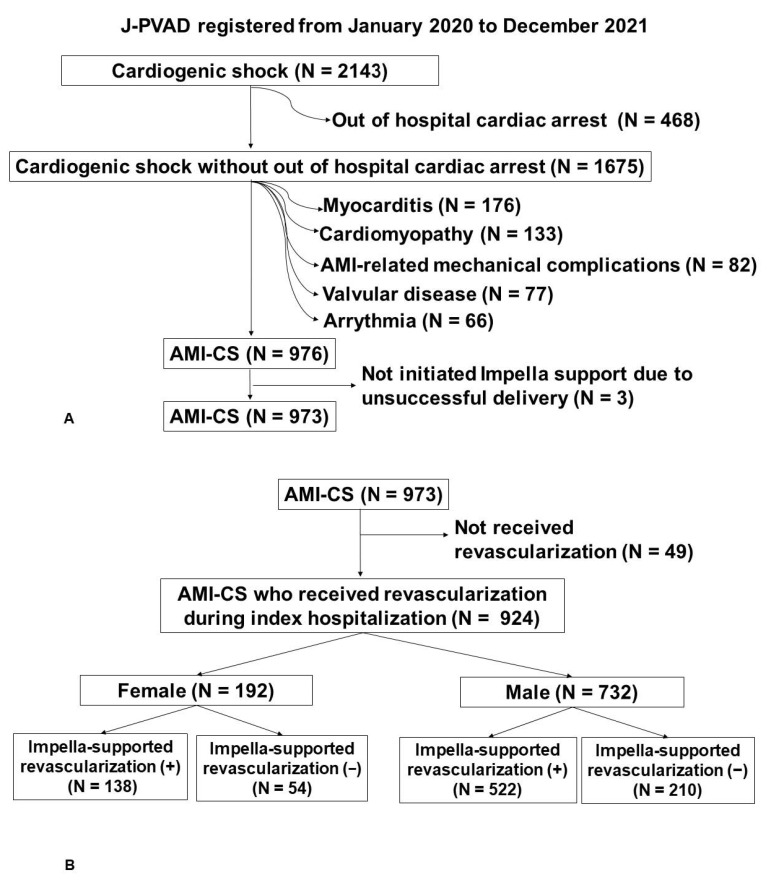
Patient population of AMI-CS was shown (**A**). Patients with AMI-CS who received revascularization during index hospitalization were included in this study (**B**). AMI-CS, Acute myocardial infarction-related cardiogenic shock; AMI, acute myocardial infarction.

Inclusion and exclusion criteria are summarized in Figure 1A,B. Of all patients, those who underwent successful Impella implantation for the treatment of AMI-CS between January 2020 and December 2021 were included in this analysis. Patients with out-of-hospital cardiac arrest were excluded given the significant impact of pre-hospital cardiopulmonary resuscitation on mortality [11]. Patients without revascularization during the index hospitalization were excluded because of the clear survival benefit of early revascularization [12]. Patients with AMI-related mechanical complications, such as ventricular septal rupture, were excluded given the completely different purpose for the use of the Impella device and treatment strategy, including surgical repair operation. 

This study was also approved by the local institutional review board beforehand (IRB number R2020076). Written informed consent was wavered due to the retrospective nature and opt-out of this study. 

### 2.2. Impella Device

Several types of Impella devices have been used to treat ACS-CS, depending on the era, underlying disease, hemodynamics, access vessel size, and estimated support duration. Impella 2.5 and 5.0 have been commercially available for the treatment of drug-resistant acute heart failure (AHF) since September 2017, and Impella CP has been used since July 2019. Impella 5.5 has been available since April 2022. The Impella 5.5 was not included in this P-VAD registry due to the registration period. IABP was used before and/or after Impella support. Veno-arterial ECMO was also used before, during, and/or after Impella support at the discretion of each center.

### 2.3. Collected Data

Baseline characteristics including comorbidities, concomitant procedures, and laboratory data were collected at baseline (just prior to Impella placement). Intravenous inotropes administered at the time of Impella removal, temporary MCS devices placed before Impella placement and added or converted after Impella support, and New York Heart Association (NYHA) classifications at discharge and 30 days after Impella removal were collected. Left ventricular ejection fraction (LVEF) on echocardiography at baseline and after Impella removal were retrieved. Adverse events (e.g., bleeding and hemolysis) occurring during and after Impella support were also collected.

### 2.4. Endpoints

The independent variable was defined as female sex versus male sex. The primary endpoint was 30-days survival after the initiation of Impella support. The secondary endpoint was the occurrence of Impella-related adverse events.

### 2.5. Statistical Assessments

Statistics were performed using JMP pro ver14.0 (SAS Institute Japan. Tokyo, Japan). Variables with *p* < 0.05 were considered statistically significant. Continuous data were described as median and interquartile ranges and compared between two groups using Mann–Whitney U test. Categorical data were displayed as numbers and percentage and compared between two groups by Chi-square test or Fischer’s exact test as appropriate. 

The 30-day survivals after initiation of Impella support were compared between female and male by the Kaplan–Meier analyses and the log-rank test. The impact of female sex on the 30-day mortality was investigated by the Cox proportional hazard ratio regression analyses. The five lowest variables with *p* < 0.05 in the univariable analyses, including female sex irrespective of its *p* value, were included in the multivariable analyses. 

## 3. Results

### 3.1. Patient Selection Flow

A total of 2143 patients with CS who received Impella support and were registered in the J-PVAD registry between January 2020 and December 2021 were eligible (Figure 1A). Of these, 468 patients with out-of-hospital cardiac arrest and 699 patients with etiologies other than AMI-CS were excluded. Three patients without successful Impella placement were excluded. We further excluded 49 patients who did not receive revascularization during index hospitalization. Finally, we included 924 patients with AMI-CS who received revascularization during index hospitalization (Figure 1B). 

### 3.2. Baseline Characteristics

A total of 924 patients were included (Table 1). All patients had AMI-CS and underwent invasive coronary artery revascularization. Median age was 73 (64, 79) years and 192 (21%) were female. Median systolic blood pressure was 89 (72, 107) mmHg. Median left ventricular ejection fraction was 30% (20%, 40%). Median lactate level was 4.4 (2.5, 7.8) mmol/L. Most of the patients (72.4%) had received intravenous inotropes before Impella support. Ninety-six (10.4%) patients had received IABP and 178 (19.4%) patients received ECMO support before Impella placement. 

There were several differences in baseline characteristics between female and male patients (Table 1). The female patients were older and had smaller physiques than the male patients (*p* < 0.05 for both). Median LVEF was higher in the female patients. The prevalence of intravenous inotropes users was higher in female patients than the male patients. There was no significant difference between female and male patients concerning the prevalence of supraventricular and ventricular arrhythmias. The prevalence of IABP and ECMO support before Impella placement was also not significantly different between female and male patients.

### 3.3. Impella Device and Concomitant Procedure

Treatment data are displayed in Table 2. As detailed above, several types of Impella were used (2.5, CP, and 5.0). Most of the patients (92.8%) received Impella CP. Most of the revascularization (97.1%) was performed by percutaneous coronary intervention (PCI) and others (9.1%) were by coronary artery bypass grafting. 

Impella support was initiated prior to revascularization in most of the patients (71.4%): they received revascularization under Impella support. Others (28.6%) initiated Impella support after revascularization: they received revascularization without Impella support. First Impella support was continued during 3.8 (1.8, 6.4) days on median. 

Several therapeutic parameters were different between female and male patients (Table 2). The female patients received Impella 2.5 as the first Impella more frequently (i.e., smaller device) than the male patients. The type of revascularization was not significantly different. The timing of Impella support initiation (i.e., before versus after revascularization) was not different. The duration of first and second Impella support was shorter in the female patients. The incidence of structural heart disease intervention during index hospitalization (i.e., transcatheter mitral valve edge-to-edge repair) was significantly higher in female patients. The incidence of cardioversion as well as electrophysiological study/ablation were not significantly different between female and male patients.

### 3.4. Impella Removal

Following revascularization with/without Impella support, Impella was successfully removed in 574 (62.1%) patients (Table 3). Others (37.9%) died on Impella supports. A few patients initiated IABP and ECMO support after Impella removal (5.4% and 1.9%, respectively; Table 3). Very few patients (0.3%) were converted to durable LVAD. 

The rate of successful Impella removal tended to be lower in the female patients (*p* = 0.0760). The prevalence of patients receiving inotropes, IABP, ECMO, and durable LVAD was not significantly different between the two groups. 

### 3.5. Primary Outcome

**Figure 2:**
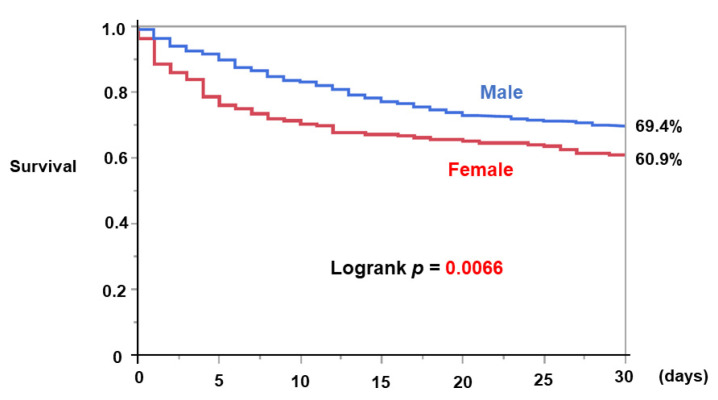
Comparison of 30-day survival between female and male patients after initiation of Impella support in AMI-CS was shown. AMI-CS, Acute myocardial infarction-related cardiogenic shock.

The 30-day survival of all patients following the initiation of Impella support, which was a primary outcome, was 67.6%. It was significantly lower in female patients than in male patients (60.9% versus 69.4%, *p* = 0.0066; Figure 2). Female sex was significantly associated with the primary outcome, adjusted for the other four potential confounders that were significant in the univariable analyses, including the initiation of Impella before revascularization with a hazard ratio of 1.365 (95% confidence interval 1.026–1.816, *p* = 0.0324; Table 4).

**Figure 3:**
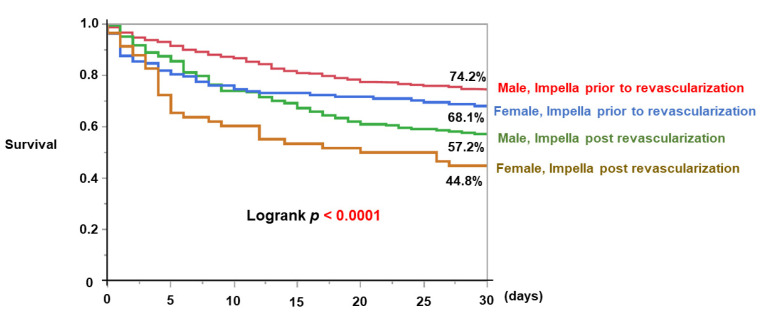
Comparison of 30-day survival between female and male patients with or without Impella-supported revascularization in AMI-CS was shown. AMI-CS, Acute myocardial infarction-related cardiogenic shock.

Among the female patients, 138 patients received Impella support before revascularization. The female patients who initiated Impella before revascularization had significantly higher 30-day survival compared to the female patients who initiated Impella after revascularization (68.1% versus 44.8%, *p* = 0.0015; Figure 3).

The 30-day survival after Impella removal was also significantly lower in female patients (57.3% versus 66.8%, *p* = 0.0173; Table 5). The survival discharge tended to be lower in female patients (52.1% versus 61.5%, *p* = 0.0514). 

### 3.6. Secondary Outcome

Adverse events are summarized in Table 6. Among adverse events during index hospitalization, there was no significant difference between female and male on the occurrence of bleeding, hemolysis, sepsis and local infection, aortic valve regurgitation, and cerebrovascular accident. However, the incidence of lower limb ischemia was significantly higher in female patients compared to male (8.9% versus 4.1%, *p* = 0.014).

## 4. Discussion

In this study, we evaluated the sex difference in patients with AMI-CS receiving Impella support in the short-term prognosis using multicenter, large-scale, J-PVAD registry data. Female patients had significantly lower 30-day survival and a higher incidence of lower limb ischemia compared to male patients. Female patients who initiated Impella support prior to revascularization had better outcomes than female patients who initiated Impella support after revascularization. 

### 4.1. Sex Differences in Patients with AHF

Unique sex differences in the clinical phenotype of patients with AHF have been well-documented in previous studies [13]. Female patients with AHF are older and more likely to have hypertension, atrial fibrillation, valvular heart disease, diabetes, anemia, and depression [14]. In our data, female patients were older and tended to have a history of hypertension, whereas the incidence of atrial and ventricular arrhythmia was not significantly different from male patients. 

Echocardiographic study demonstrated that the hospitalized female patients with AHF had higher LVEF, tricuspid annular plane systolic excursion, and measures of filling pressures (median E/e’), despite similar baseline pulmonary congestion compared to male patients [15]. In our data, the LVEF was significantly higher in female patients, while intravenous inotropes were administered at a higher rate in female patients. 

The incidence of structural heart disease intervention such as transcatheter mitral valve edge-to-edge repair was significantly higher in female patients. Severe mitral regurgitation secondary to papillary muscle tear is a rare but overwhelmingly morbid and often fatal complication of acute myocardial infarction [16], and case reports of successful transcatheter edge-to-edge repair for acute mitral regurgitation associated with AMI-CS have recently been reported [17]. Female patients may present with more complex AMI-CS associated with functional mitral regurgitation. 

Given the lack of significant difference in the incidence of cardioversion and electrophysiological study/ablation during index hospitalization between female and male patients, there may be no sex differences in arrhythmias in patients with AMI-CS receiving Impella support and revascularization. However, we have excluded patients with out-of-hospital cardiac arrest in this study and there may be a selection bias. We do not have data on the incidence of new-onset atrial fibrillation. 

### 4.2. Sex Differences in Patients with AMI-CS

AMI is the most common precipitating factor of AHF [18]. Of these, CS is the most severe and fatal form [19]. Reported sex differences in this cohort are controversial [4,6,19,20]. Female patients are generally older and have more advanced renal failure, while they less frequently have a history of ischemic heart disease and smoking [19,21]. Female patients present more advanced forms of shock, whereas the lactate level and the degree of multi-organ dysfunction are comparable to male patients [21]. 

Our cohort had almost comparable results, except for having advanced renal failure at baseline. Of note, female patients had more elevated C-reactive protein and more advanced hypoalbuminemia than the male patients. Lactate level in the female patients was comparable to the male one. Systemic inflammation and malnutrition status, in addition to peripheral hypoperfusion indicated by lactate level, may be major contributors of lower short-term survival in female patients receiving Impella support [2]. 

### 4.3. Impact of the Female Sex with MCS Therapy on the Short-Term Prognosis

A sub-analysis of the CULPRIT-SHOCK trial and the report from Spanish National Health System Minimum Basic Data described how female patients with CS who received MCS had higher in-hospital mortality than male patients [19,22]. One study found that female patients had less chance to receive MCS than male patients [20]. Another study demonstrated that the in-hospital mortality of female patients receiving ECMO was comparable to those of male patients when baseline characteristics were appropriately adjusted [23]. In our study, the negative prognostic impact of female sex still remained after the adjustment for baseline characteristics, including smaller physiques. 

On the other hand, female patients who initiated Impella support before revascularization (i.e., they received Impella-supported revascularization) had greater survival than females who received Impella after revascularization. The positive prognostic impact of Impella-supported revascularization was comparable with male patients. In the previous study, PCI under Impella support was associated with greater survival benefits in female patients [3]. In another study, female patients rarely received PCI and MCS compared to male patients, resulting in higher in-hospital mortality [14]. In another study, Impella initiation prior to PCI was associated with significant reduction in inotrope use and may be associated with improved mortality and morbidity in patients with AMI-CS in both genders [24]. Initiation of Impella support prior to coronary artery revascularization may be the key to improving survival in female patients with AMI-CS.

In the cVAD Registry from the US, female patients had more critical vascular complications [3]. In our study, female patients had lower limb ischemia more frequently than male patients, probably due to their smaller vessel size. These complications may have helped terminate Impella support earlier. On the contrary, the incidence of MCS therapies initiated after Impella removal was not different between females and males. Given the negative prognostic impact of body mass index in our study, the achievement of recovery of cardiac function as well as end-organ dysfunction might have been obtained earlier in female patients due to their small physiques and oxygen demand. The incidence of bleeding complications was not significantly different between female and male patients in this study, whereas the bleeding complications in patients with ST elevation myocardial infarction as well as patients who underwent high-risk PCI with MCS are reported to be more common in female patients [25,26]. Relatively early removal of Impella in female patients compared to males might have reduced the incidence of bleeding complications. The pulmonary artery catheter was less used in female patients in our study. This may also be due to their small physiques, whereas the use of a pulmonary artery catheter had no significant impact for primary outcome in our study. 

### 4.4. Study Limitations

This was an observational registry data, including heterogeneous patient population. No standardized AHF treatment protocols were used across participating centers. The indication and timing of Impella support were completely at the discretion of the attending clinicians. Due to the nature of the registry design and an ethical issue, there was no control arm not receiving Impella. Due to the nature of multicenter registry, several data including echocardiographic and laboratory data (e.g., maximum serum creatinine kinase levels, high-sensitivity cardiac troponin T levels, serum N-terminal pro B-type natriuretic peptide levels, and serum low-density lipoprotein cholesterol levels) are lacking. The culprit legion and SYNTAX Score had not been collected and the information of the coronary artery is missing in this study. In addition, the information of the antiplatelet/anticoagulant therapy is also lacking. We also had no information concerning COVID-19 infection, which may have affected the prognosis in patients with AMI-CS.

## 5. Conclusions

Regarding female patients with AMI-CS receiving Impella support and who underwent revascularization, female sex was significantly associated with lower 30-day survival. The initiation of Impella support before revascularization may be recommended to achieve greater survival. Further investigation is needed to verify the prognostic impact of female sex for patients with AMI-CS receiving Impella support on short-term mortality.

## Figures and Tables

**Table 1:** Baseline characteristics.

	All Patients (n = 924)	Female (n = 192, 21%)	Male (n = 732, 79%)	*p* Value
Demographics				
Age (years)	73 (64, 79)	78 (72, 84)	71 (62, 78)	<0.0001 *
Hypertension	618 (66.9%)	135 (70.3%)	483 (66.0%)	0.1036
Diabetes mellitus	407 (44.1%)	84 (43.8%)	323 (44.1%)	0.9104
Dyslipidemia	462 (50.0%)	92 (47.9%)	370 (50.6%)	0.3044
Pulmonary hypertension	23 (2.5)	6 (3.1%)	17 (2.3%)	0.4967
Cerebrovascular accident	76 (8.2%)	20 (10.4%)	56 (7.7%)	0.4007
Smoking	515 (55.7%)	34 (17.7%)	481 (65.7%)	<0.0001 *
Atrial fibrillation/tachycardia	52 (5.7%)	12 (6.3%)	40 (5.5%)	0.7247
Ventricular tachycardia/fibrillation	20 (2.2%)	4 (2.1%)	16 (2.2%)	1.0000
Body mass index (kg/m^2^)	23.3 (20.7, 25.9)	22.4 (19.6, 25.4)	23.4 (21.0, 26.0)	0.0045 *
Body surface area (m^2^)	1.66 (1.52, 1.81)	1.43 (1.33, 1.56)	1.72 (1.59, 1.84)	<0.0001 *
Intravenous inotropes before Impella	669 (72.4%)	150 (78.1%)	519 (70.9%)	0.0465 *
Intra-aortic balloon pump	96 (10.4%)	17 (8.9%)	79 (10.8%)	0.5070
Extracorporeal membrane oxygenation	178 (19.4%)	35 (18.3%)	143 (19.6%)	0.7578
Systolic blood pressure (mmHg)	89 (72, 107)	91 (71, 110)	89 (72, 106)	0.5065
Diastolic blood pressure (mmHg)	59 (45, 72)	58 (45, 73)	59 (45, 72)	0.5040
Heart rate (bpm)	91 (72, 110)	90 (71, 106)	92 (72, 110)	0.2674
Echocardiographic data				
LVEF before Impella insertion (%)	30 (20, 40)	35 (26, 44)	30 (20, 35)	<0.0001 *
Laboratory data				
Lactate (mmol/L)	4.4 (2.4, 7.8)	5.0 (2.7, 8.5)	4.2 (2.3, 7.5)	0.0591
Creatinine kinase (IU/L)	250 (109, 869)	369 (121, 948)	234 (108, 853)	0.2386
Total bilirubin (mg/dL)	0.7 (0.5, 1.0)	0.6 (0.5, 1.0)	0.7 (0.5, 1.0)	0.4963
Serum Creatinine (mg/dL)	1.17 (0.92, 1.62)	0.96 (0.78, 1.39)	1.21 (0.97, 1.67)	<0.0001 *
C-reactive protein (mg/dL)	0.40 (0.10, 3.10)	0.61 (0.14, 3.47)	0.35 (0.10, 2.90)	0.0078 *
Lactate dehydrogenase (U/mL)	342 (223, 637)	391 (262, 711)	323 (216, 604)	0.0067 *
Albumin (mg/dL)	3.6 (3.1, 4.0)	3.5 (3.1, 3.8)	3.7 (3.2, 4.0)	0.0002 *
Aspartate Aminotransferase (IU/L)	57 (30, 179)	80 (36, 217)	53 (30, 168)	0.0102 *
Alanine Aminotransferase (IU/L)	35 (20, 74)	32 (18, 103)	35 (21, 68)	0.9314

LVEF, left ventricular ejection fraction. * *p* < 0.05 by Mann–Whitney U test or Fisher’s exact test as appropriate.

**Table 2:** Therapies during index hospitalization and additional MCS after first Impella placement.

	All Patients (n = 924)	Female (n = 192, 21%)	Male (n = 732, 79%)	*p* Value
Device types of first Impella				
Impella 2.5	47 (5.1%)	20 (10.4%)	27 (3.7%)	0.0006 *
Impella CP	857 (92.8%)	168 (87.5%)	689 (94.1%)	0.0028 *
Impella 5.0	20 (2.2%)	4 (2.1%)	16 (2.2%)	1.0000
Access site of first Impella				
Transfemoral approach	898 (97.2%)	187 (97.4%)	711 (97.1%)	1.0000
Subclavian/axillary approach	22 (2.4%)	5 (2.6%)	17 (2.3%)	0.7920
Use of pulmonary artery catheter	570 (61.7%)	114 (59.4%)	456 (62.3%)	0.0185 *
Cardioversion	203 (22.0%)	36 (18.8%)	167 (22.8%)	0.2412
Non-pharmacological therapy during index hospitalization				
Percutaneous coronary intervention	897 (97.1%)	187 (97.4%)	710 (97.0%)	1.0000
Coronary artery bypass grafting	84 (9.1%)	14 (7.3%)	70 (9.6%)	0.3978
Valve surgery	19 (2.1%)	5 (2.6%)	14 (1.9%)	0.5678
Impella-supported revascularization	660 (71.4%)	138 (71.9%)	522 (71.3%)	0.9286
Other surgical operation	37 (4.0%)	10 (5.2%)	27 (3.7%)	0.4064
Balloon aortic valvuloplasty	0 (0.0%)	0 (0.0%)	0 (0.0%)	
Transcatheter SHD intervention	9 (1.0%)	5 (2.6%)	4 (0.6%)	0.0244 *
EP/ablation	14 (1.5%)	3 (1.6%)	11 (1.5%)	1.0000
Additional MCS therapy during index hospitalization				
ECMO	309 (33.4%)	59 (30.7%)	250 (34.2%)	0.3911
ECMO initiated during first Impella support	101 (11.1%)	21 (11.1%)	80 (11.1%)	1.0000
Left ventricular assist device	5 (0.5%)	2 (1.0%)	4 (0.4%)	0.2786
Second Impella	57 (6.2%)	10 (5.2%)	47 (6.4%)	0.6158
Device type of second Impella				
Impella 2.5	1 (2.4%)	1 (16.7%)	0 (0%)	0.1429
Impella CP	28 (50.9%)	6 (66.7%)	22 (47.8%)	0.4688
Impella 5.0	26 (47.3%)	2 (22.2%)	24 (52.2%)	0.1485
Support duration of first Impella (days)	3.8 (1.8, 6.4)	3.5 (1.1, 6.1)	3.9 (1.9, 6.7)	0.0145 *
Support duration of second Impella (days)	6.9 (4.0, 11.6)	2.2 (0.3, 6.0)	8.8 (4.8, 12.7)	0.0009 *

SHD, structural heart disease; MCS, mechanical circulatory support; EP, electrophysiological study; ECMO, extracorporeal membrane oxygenation. * *p* < 0.05 by Mann–Whitney U test or Fisher’s exact test as appropriate.

**Table 3:** Clinical data at Impella removal and MCS after Impella removal.

	All Patients (n = 924)	Female (n = 192, 21%)	Male (n = 732, 79%)	*p* Value
Intravenous inotropes at Impella removal	647 (70.1%)	129 (67.2%)	518 (70.9%)	0.3307
Dobutamine	469 (50.8%)	90 (46.9%)	379 (51.8%)	0.2562
Dopamine	69 (7.5%)	13 (6.8%)	56 (7.7%)	0.7594
Noradrenaline	404 (43.7%)	77 (40.1%)	327 (44.7%)	0.2880
PDEIII inhibitors	57 (6.2%)	11 (5.7%)	46 (6.3%)	0.8673
Adrenaline	46 (5.0%)	9 (4.7%)	37 (5.1%)	1.0000
Vasopressin	29 (3.1%)	6 (3.1%)	23 (3.1%)	1.0000
Vital signs				
SBP at Impella removal (mmHg)	99 (64, 114)	95 (0, 114)	100 (77, 114)	0.1030
DBP at Impella removal (mmHg)	53 (32, 73)	50 (0, 60)	54 (40, 64)	0.0024 *
Heart rate at Impella removal (bpm)	80 (54, 93)	76 (0, 94)	81 (60, 93)	0.0523
Alive at Impella removal	574 (62.1%)	106 (55.2%)	468 (63.9%)	0.0760
LVEF at Impella removal (%)	40 (30, 48)	42 (35, 52)	40 (30, 48)	0.0060 *
MCS therapy after Impella removal				
Upgrade to durable LVAD	3 (0.3%)	1 (0.5%)	2 (0.3%)	0.5041
Addition of IABP	49 (5.4%)	8 (4.2%)	41 (5.7%)	0.4547
Addition of ECMO	17 (1.9%)	5 (2.6%)	12 (1.7%)	0.3703

SBP, systolic blood pressure; DBP. diastolic blood pressure; LVEF, left ventricular ejection fraction; MCS, mechanical circulatory support; LVAD, left ventricular assist device; IABP, intra-aortic balloon pump; ECMO, extracorporeal membrane oxygenation. * *p* < 0.05 by Mann–Whitney U test or Fisher’s exact test as appropriate.

**Table 4:** Univariable and multivariable analyses for the 30-day mortality.

	Univariable Analyses		Multivariable Analyses	
	Hazard Ratio (95% CI)	*p*-Value	Hazard Ratio (95% CI)	*p*-Value
Age (years old)	1.015 (1.005–1.025)	0.0033 *	1.025 (1.013–1.038)	<0.0001 *
Female sex	1.407 (1.094–1.809)	0.0077 *	1.365 (1.026–1.816)	0.0324*
Smoking	0.963 (0.753–1.233)	0.7662		
Body mass index (kg/m^2^)	1.055 (1.029–1.080)	<0.0001 *	1.057 (1.030–1.085)	<0.0001 *
Impella 2.5	0.840 (0.472–1.435)	0.5235		
Impella CP	0.791 (0.544–1.152)	0.2251		
PCI during index hospitalization	0.616 (0.435–0.870)	0.0060 *		
CABG during index hospitalization	0.598 (0.384–0.931)	0.0277 *		
Transcatheter SHD intervention	0.271 (0.038–1.932)	0.1929		
EP/ablation during index hospitalization	0.563 (0.181–1.757)	0.3230		
ECMO use before Impella placement	2.218 (1.732–2.840)	<0.0001 *	2.517 (1.967–3.221)	<0.0001 *
Pulmonary artery catheter use	0.968 (0.767–1.222)	0.7873		
LVEF at baseline (%)	0.985 (0.973–0.998)	0.0212 *		
Serum Creatinine (mg/dL)	1.009 (0.998–1.015)	0.0970		
Total bilirubin (mg/dL)	1.134 (1.064–1.193)	0.0005 *		
C-reactive protein (mg/dL)	1.038 (1.021–1.054)	<0.0001 *		
Creatinine kinase (IU/L)	1.00004 (1.00002–1.00006)	0.0001 *		
Albumin (mg/dL)	0.609 (0.517–0.720)	<0.0001 *		
Impella-supported revascularization	0.496 (0.398–0.617)	<0.0001 *	0.605 (0.473–0.775)	<0.0001 *

PCI, percutaneous coronary intervention; CABG, coronary artery bypass grafting; BAV, balloon aortic valvuloplasty; SHD, structural heart disease; EP, electrophysiological study; ECMO, extracorporeal membrane oxygenation; LVEF, left ventricular ejection fraction. * *p* < 0.05 by logistic regression analysis.

**Table 5:** Clinical outcomes following the initiation of Impella support.

	All patients (n= 924)	Female (n = 192, 21%)	Male (n = 732, 79%)	*p* Value
30-day survival after Impella removal	599 (64.8%)	110 (57.3%)	489 (66.8%)	0.0173
NYHA classification at 30 days after Impella removal				0.0503
Class I	190 (24.0%)	33 (20.8%)	157 (24.8%)	
Class II	206 (26.0%)	36 (22.6%)	170 (26.8%)	
Class III	88 (11.1%)	13 (8.2%)	75 (11.8%)	
Class IV	309 (49.0%)	77 (48.4%)	232 (36.6%)	
Survival discharge	550 (59.5%)	100 (52.1%)	450 (61.5%)	0.0514
NYHA classification at discharge				0.0047
Class I	200 (25.0%)	43 (25.9%)	157 (24.8%)	
Class II	237 (29.7%)	35 (21.1%)	202 (31.9%)	
Class III	54 (6.8%)	7 (4.2%)	47 (7.4%)	
Class IV	308 (38.6%)	81 (48.8%)	227 (35.9%)	

NYHA, New York Heart Association.

**Table 6:** Adverse events following the initiation of Impella support.

	All Patients (n = 924)	Female (n = 192, 21%)	Male (n = 732, 79%)	*p* Value
Adverse events due to Impella device	55 (6.0%)	13 (6.8%)	42 (5.8%)	0.6070
Adverse events during index hospitalization	595 (64.4%)	135 (70.3%)	460 (62.8%)	0.1191
Bleeding (including hematoma)	243(26.3%)	53 (27.6%)	190 (26.0%)	0.646
Sepsis and local infection	73 (7.9%)	19 (9.9%)	54 (7.4%)	0.2917
Hemolysis	156 (16.9%)	29 (15.1%)	127 (17.4%)	0.5166
Aortic valve regurgitation	8 (0.9%)	3 (1.6%)	5 (0.7%)	0.3737
Cerebrovascular accident	52 (5.6%)	12 (6.3%)	40 (5.5%)	0.7247
Renal failure	93 (10.1%)	18 (9.4%)	75 (10.3%)	0.7887
Lower limb ischemia	47 (5.1%)	17 (8.9%)	30 (4.1%)	0.0148
Vascular injury requiring intervention	18 (2.0%)	4 (2.1%)	14 (1.9%)	0.7761
Thrombocytopenia	68 (7.4%)	10 (5.2%)	58 (7.9%)	0.2181
Thrombotic complications without cerebrovascular accident	12 (1.3%)	4 (2.1%)	8 (1.1%)	0.2854

## Data Availability

The deidentified participant data will not be shared.
